# Understanding the Impact of the COVID-19 Pandemic on the Perception and Use of Urban Green Spaces in Korea

**DOI:** 10.3390/ijerph20043018

**Published:** 2023-02-09

**Authors:** Jiwon Kim, Youngjin Ko, Whijin Kim, Gaeun Kim, Jeongmin Lee, Olebogeng Thelma G. Eyman, Sarwat Chowdhury, Julie Adiwal, Yowhan Son, Woo-Kyun Lee

**Affiliations:** 1 Department of Environmental Science and Ecological Engineering, Korea University, Seoul 02841, Republic of Korea; 2 United Nations Development Programme Seoul Policy Centre, Seoul 02841, Republic of Korea

**Keywords:** urban green space (UGS), landscape planning, resilience, nature-based solutions (NbS), COVID-19

## Abstract

Faced with the prospect that the impact of the COVID-19 pandemic and climate change will be far-reaching and long-term, the international community is showing interest in urban green space (UGS) and urban green infrastructure utilization as a solution. In this study, we investigated how citizens’ perceptions and use of UGS have changed during COVID-19. We also collected their ideas on how UGS can raise its usability. As a result, more people became to realize the importance of UGS. In particular, the urban environmental purification function from UGS was recognized as giving great benefits to respondents. On the other hand, the patterns of UGS use were mixed with decreasing UGS use to maintain social distancing or increasing UGS use to maintain health or substitute other restricted facilities. More than half of respondents had their UGS visit patterns impacted by COVID-19. In particular, the increase rate of UGS use was rather high in the group that seldom used UGS before COVID-19. In addition, they increased the use of UGS to replace other limited facilities, and thus tended to demand an increase in rest facilities. Based on these results, this paper suggested securing social support and sustainability for the policy by reflecting users’ demand in landscape planning related to the increase of UGS in the city. This study can contribute to improving the resilience of UGS and the sustainability of urban space planning.

## 1. Introduction

### 1.1. Urban Green Space as the Source of Ecosystem Services

With growing urbanization, the number of people living in urban areas is increasing. As more and more people live in cities, urban green spaces (UGS) have received growing attention for their environmental benefits due to the emerging demands of urban society for coping with various environmental or health problems, and building more sustainable cities [1,2]. According to the United Nations Sustainable Development Goal 11 (SDG 11) “sustainable cities and communities”, creating public green spaces is one way to make cities sustainable [3]. Following this trend, the proper creation of UGS has been given importance in terms of urban planning [4].

The benefits from UGS can be called “ecosystem services”, which are defined as benefits that people can derive directly or indirectly from an ecosystem [5,6]. The ecosystem services from UGS can enhance both the physical and psychological wellbeing of citizens [7]. For example, UGS plays an important role in supplying regulating services such as by mitigating intense heat in urban areas [8]. Urban vegetation can sequestrate carbon dioxide through photosynthesis processes and decrease levels of air pollutants services [9]. Additionally, it contributes to solving various problems by generating services such as rainwater drainage and mitigating risks from flooding [10]. In addition, UGS improves the quality of urban life by providing people places for physical activities social networking, reducing social isolation [11], and promoting a sense of community [12]. In addition, contact with the natural environment has positive effects on mental health and may help to reduce stress [13,14].

### 1.2. The COVID-19 and UGS

The COVID-19 pandemic began in early 2020 and has had an enormous impact not only on global public health, but also on economic, social, and environmental sectors. As the COVID-19 is spread by human-to-human contact [15], densely populated urban spaces have a higher contagion rate of COVID-19. To decelerate the spread of infection, border closures, regional lockdowns, and social distancing—government regulations keeping people physically distant indoors—were employed in most countries [16]. Lockdown and social distancing changed people’s lifestyles, and caused largest secondary impacts, apart from the disease itself, such as social isolation, increased stress, and negative socioeconomic effects [17]. However, at the same time, this situation provided an opportunity to make people aware of the importance of UGS [18,19]. During lockdown and restrictions, to deal with these psychological distress and mental health issues, UGS has become one of the important sources of resilience for people, especially in urban areas. UGS replaced various outdoor activities due to their accessibility and availability as gathering places for small numbers of people while maintaining social distancing [20]. There are two reasons UGS have become more important with time. The first reason is that UGS acts as a “spatial vaccine” in terms of physical and psychological effects. UGS provide safer open spaces than indoor/closed space for physical activities, which reinforce immune systems [21,22]. In addition, by visiting UGS, people can relieve their physical, social, and mental isolation that caused them greater stress and “corona blues” from social distancing [23].

### 1.3. Understanding Users’ Perception for Landscape Planning

Perception includes attitude [24]. Attitudes direct an individual to act or react in a particular way when faced with a situation, person, or object, based on his/her experience and moral standards [25]. Therefore, the user’s perception is closely related to the promotion of the use of the urban landscape. Recently, users’ awareness of urban space has been positioned as being of prime importance in urban landscape management [26]. When making decisions on public management, considering users’ reactions to public services is emphasized [27], and New Public Governance was suggested, where users of public services actively participate in decision-making related to policy design [28].

Therefore, understanding the perceptions of urban residents on public green infrastructure is fundamental in urban landscape planning [29]. In other words, when the decision regarding UGS is made, the local people’s perceptions and behaviors on UGS have to be fully understood and considered in order to improve the environmental quality of cities [30,31].

### 1.4. Aim and Theoretical Framework

As COVID-19 had unprecedented impacts on the global society, research regarding the importance of UGS during a pandemic has been actively conducted [32,33,34,35,36]. Nevertheless, survey-based research among urban residents is rarely utilized [37]. Moreover, although UGS in megacities with higher population densities, such as Beijing, Seoul, or New York City, are predicted to experience a greater impact from a pandemic [36], there are a not many related studies focused on these cities [38,39]. This study aims to fill this research gap based on the hypothesis that COVID-19 and social distancing may have changed people’s perception and usage of public green spaces [20]. Understanding this impact can increase the applicability of nature-based solutions in cities and enhance the sustainability of landscape planning through increased use and awareness of green spaces [40,41]. In addition, analyzing in relation to corona can increase the resilience of green spaces to disturbances such as pandemics, and to support developing mid- to long-term policy for the creations of urban green spaces. In this study, the awareness and using pattern of Korean urban residents on UGS before and after COVID-19 were surveyed and the patterns and causes of changes in the perception of UGS were identified. Through this survey, the implications for public landscape planning utilization of UGS for improving the resilience and adaptive capacity to cope with future disturbances were derived.

## 2. Materials and Method

### 2.1. Study Area

**Figure 1:**
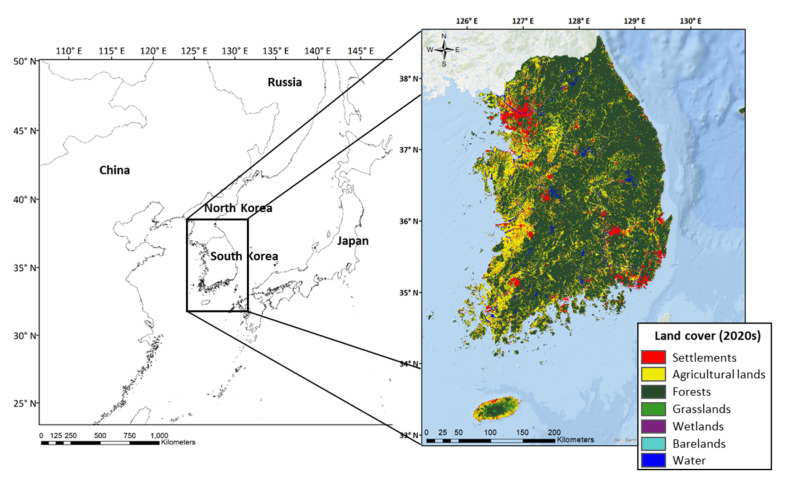
The location and land cover map of the study area.

South Korea (Korea) is one of the countries struggling to foster UGS for nature-based solutions (NbS). In Korea, as a regional urban system centered on large cities is formed, there is growth of big cities in the metropolitan area and rapid decline of small and medium-sized cities in the metropolitan area [42]. In fact, Korea is one of the most densely populated countries in the world, where about 84.41% of the total population live in urban areas, which is only about 15.96% of total area in 2020 according to the World Bank and Korea Land and Geospatial Informatix Corporation (Figure 1). In particular, Seoul, the capital of Korea, is drawing attention as one of the world’s megacities [43]. As urbanization and industrialization have become more serious, problems such as biodiversity loss, habitat fragmentation, and worsening environment quality have occurred [44].

The Korean government is applying NbS as a way to overcome these urban environmental problems. This is based the successful experience of restoring forests devastated after World War II and recording world-class forest stock through excellent forest management [45]. They are systematically creating and managing UGS, such as enacting the Act on Urban Parks and Green Spaces, and requiring each local government to establish a 10-year basic park and green space plan. As well as the government, the Korean people are aware of the importance of forests and green spaces. As a result of the public awareness survey on forests conducted by the Korea Forest Service in 2015, nine out of ten general citizens and experts answered that “forests and trees are related to our daily life” [46]. In addition, the “2020 Quality of Life Report” released by the Statistical Research Institute reported that satisfaction with the natural environment, such as mountains and parks, in the residential area was about 58.7%.

**Figure 2:**
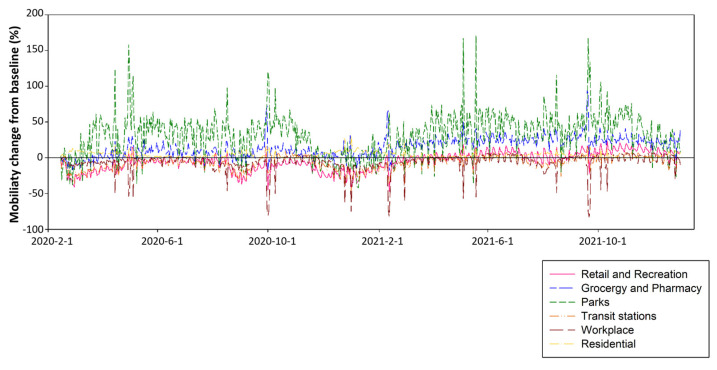
Change in mobility for parks from 15 February 2020 to 31 December 2021 in Korea (Source: modified from Google Community Mobility Reports).

Korea can be said to be one of the representative countries that actively and positively utilized UGS during COVID-19. The “COVID-19 Community Mobility Report” from 15 February 2020 to 31 December 2021, provided by Google, shows that the mobility pattern to various public spaces increased in Korea compared with the baseline, which is the normal use pattern before the outbreak of COVID-19 (Figure 2).

Mobility to most of the facilities such as retail and recreation, transit stations, and work spaces decreased or retained their normal patterns. Although the daily mobility fluctuated, the mobility to parks increased on average by 31.08% compared with normal, except when the level of social distancing enhanced to level 2.5 from December 2020 to February 2021. Sometimes, it increased over 50% to 150% compared with that prior to COVID-19.

### 2.2. Method

#### 2.2.1. Research Design and Process

The objective of the study was to understand how COVID-19 has changed citizens’ perspectives of and attitude toward green spaces and urban forests, and to figure out how to improve the usage of UGS. A survey is a commonly used method in studies on the attitudes or perceptions of people toward specific places, phenomena, or objects [47,48,49], and can be found in studies on the relationship between diseases and UGS noted in this study [50,51]. Therefore, we designed the research in the following order, namely developing the questionnaire, performing the survey, aggregating and visualizing the results, and finally drawing explanations and implications for the results. Specifically, the survey was developed to investigate (1) the users’ perception of the value of UGS, (2) whether COVID-19 affected their using pattern of UGS, (3) the impact of COVID-19 specifically on UGS visits and usage, and (4) improvements to ensure or encourage UGS utilization in the future occurrence of a pandemic. The survey was developed using a Google Form and was conducted with a total of 214 people from 12 April to 28 April 2021. The survey was conducted online and was conducted through link sharing through SNS within Korea.

#### 2.2.2. Questionnaire

The questionnaire consisted of questionnaire guidance, demographic information, respondents’ basic knowledge or attitude toward UGS, changes in UGS usage behavior before and after COVID-19, changes in UGS usage purpose, and finally, requirements to improve UGS usage. In the questionnaire guidance, the information and contact information of the person in charge and the purpose of the survey were described. In addition, the definition of UGS was revised and presented so as to help the general public understanding as a space or facility based on natural ecology that creates a high-quality urban environment and helps citizens relax and cultivate emotions [52,53]. The demographic information includes gender, age group, occupation, and workplace/work style. The items for the basic knowledge or attitude toward UGS consists of information on the types of places that people recognize as UGS, the benefits of UGS, and its importance. Afterwards, the respondents were asked about the frequency of UGS visits or the usage patterns before and after COVID-19. Finally, improvements or ways to promote of UGS were investigated. Multiple responses were allowed according to the characteristics of the question. The questionnaire was reviewed economy before the survey by experts with a background in forestry, ecology, and social science.

#### 2.2.3. Survey Analysis and Visualization

The survey results were delivered to us in Excel format through a Google Form, and we rearranged and synthesized the survey results using Excel and Sigma Plot. In this process, a table consisting of the responses collected was created, and the statistical distribution of responses was visualized with a histogram. In particular, the responses regarding the change between before and after COVID-19 were distinguished with different colors; responses before COVID-19 were marked in blue and responses after COVID-19 were marked in red. Similarly, when UGS utilization increased after COVID-19, points were displayed inside the bar graph, while the diagonal lines were inserted within the bar graph when it decreased. In addition, a transition matrix was created by counting the number of respondents of the UGS visit cycle and the importance before and after COVID-19. The transition matrix is a methodology for constructing and identifying transition matrices between different stages of a population from time t to time t + 1, and is widely used to monitor and predict environmental and ecological changes such as population and socioeconomic changes and land cover changes [54,55,56,57]. This is a table that summarizes the specific properties or regions of the study subjects collected, analyzed, and classified over two periods, and a number of summary measurements are usually derived from the change matrix [56]. In this study, the transition matrix was used for the purpose of analyzing the change between the number of respondents for a specific question in two periods, before and after COVID-19. The survey was analyzed and focused on using the developed tables and figures.

## 3. Results

### 3.1. Respondents’ Profile

A total of 214 participants responded to the survey, and the general characteristics of the respondents were as shown in Table 1.

They consist of 119 males (55.61%) and 95 females (44.39%). Although the survey was conducted regardless of age group, from teenagers to seniors, the majority of respondents were in their 20 s (38.79%) and 30 s (20.56%). Regarding occupation, students accounted for the largest portion at 30.84%, followed by office workers at 28.04% and technicians at 19.63%. The majority of respondents (52.80%) worked at their offices and 30.84% of respondents were able to work both at offices and from home. The workplace of 15 respondents who were unemployed at the time of response, except one of non-response, was assumed to be an activity space to prepare for employment.

### 3.2. The Awareness of UGS

**Figure 3:**
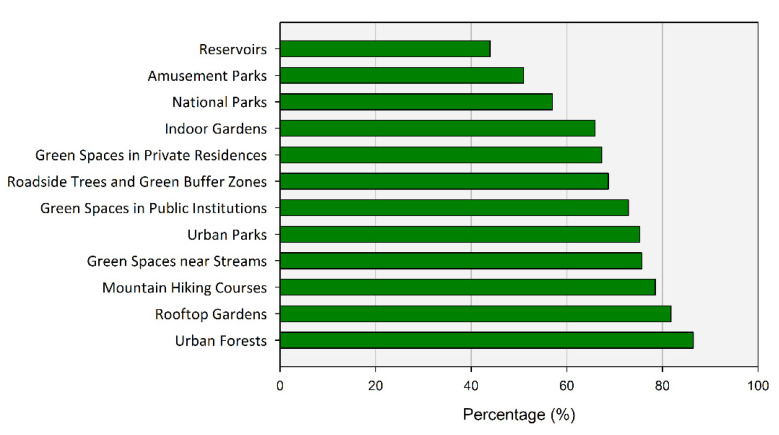
The facilities and locations that respondents recognize in the category of UGS.

At the beginning of the survey, respondents’ awareness for the types and benefits of UGS was first asked, and multiple responses were allowed. As a result of the survey, more than 80% of respondents recognized urban forest (86.45%) and rooftop garden (81.78%) as UGS (Figure 3).

Facilities created for a specific purpose, such as reservoirs or amusement parks, spaces mainly located on the outskirts of the city and/or ambiguous to include within the city such as national parks, and small-scale spaces created for landscaping purposes, such as roadside trees or residential green areas, were answered as being included in UGS by less than 70% of the respondents. In addition, indoor gardens were rarely recognized as UGS with only a 65.89% response rate.

**Figure 4:**
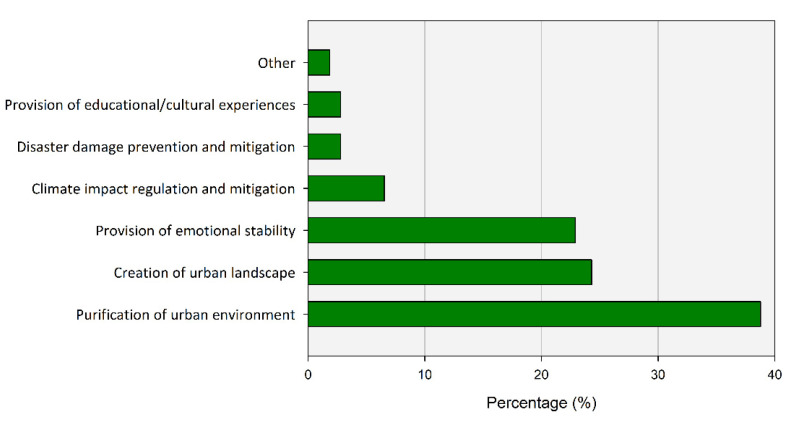
Percentage of respondents to benefit from UGS.

Regarding the question related to the benefits provided by UGS, the answer with the highest response rate was “purification of urban environment” (38.79%), such as countering air pollution and noise pollution (Figure 4).

The next responses were “fostering beautiful urban scenery” with 24.30% of the total respondents, and similarly “provision of emotional stability” with 22.90%. In addition, 1.87% of the respondents chose “other” and stated that all of the items presented corresponded to the benefits of UGS. Among the listed items, the answers corresponding to CES accounted for about 50% of the respondents. The major function that people expect from UGS is concentrated on preventing fine dust or noise, and it seems to correlate with the recent increase in public interest in air pollution such as fine dust. Considerably low response rates were recorded for climate impact control (6.54%), such as mitigation of the heat island effect, and disaster prevention/mitigation effects (2.80%), such as flooding.

**Figure 5:**
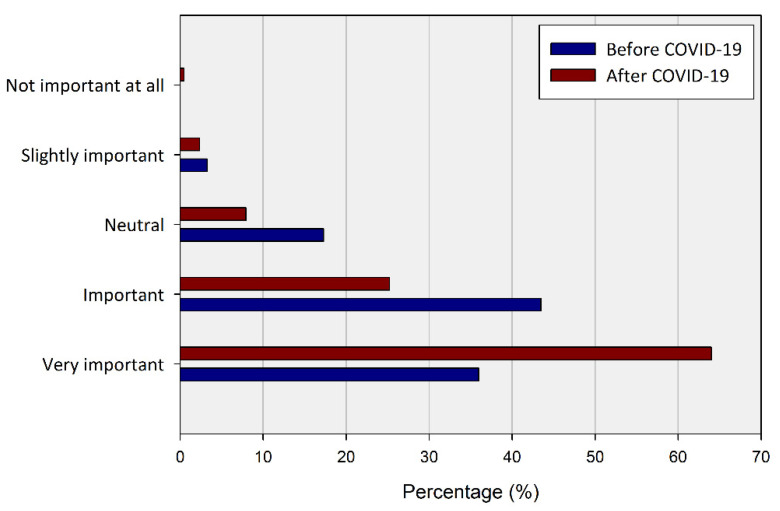
Percentage of the recognized importance of UGS before and in the wake of COVID-19.

Based on the basic perception of UGS, the frequency and purpose of visiting UGS before and after COVID-19 were asked. The change in respondents’ view on the importance of UGS before and after the outbreak of COVID-19 are shown in Figure 5 and Table 2.

The rate of recognizing the importance of UGS was 79.44% before COVID-19 and 89.25% after COVID-19, and most of the respondents were already aware of the value of UGS. About 93.53% (159 out of a total of 170 people) of respondents who thought UGS was important before COVID-19 answered that UGS are still or are more important after COVID -19. Only about 6.54% of respondents rated the importance of UGS low formerly, but 71.43% of them newly recognized the importance of UGS after COVID-19. Those who did not change their opinion about UGS, regardless of the pandemic, accounted for 57.94% of the total respondents.

### 3.3. The Change in UGS Utilization

**Figure 6:**
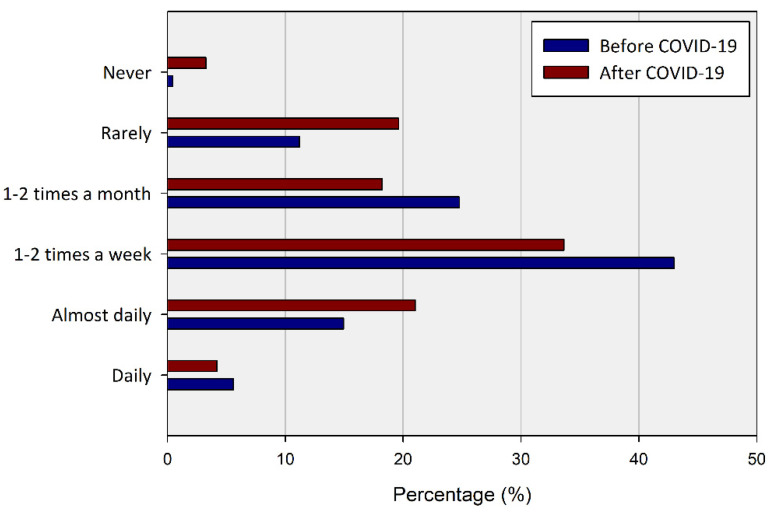
Percentage of respondents for the frequency of utilization of UGS before and in the wake of COVID-19.

Changes in the frequency of UGS utilization showed a different pattern from changes in the perception of the importance of UGS. Respondents who visited UGS more than often (daily, almost daily, or weekly) before COVID-19 accounted for 63.55% of the total, but decreased to 58.88% after COVID-19 (Figure 6 and Table 3).

In addition, the group with a high frequency of UGS use formerly decreased the frequency of use, and the low frequency group tended to diverge their utilization. About 65% of “almost daily” users maintained their use pattern, but in the case of “daily” users, half of them reduced the frequency of UGS use after COVID-19. Most of the users who visited 1–2 times a week had reduced their visits to 1–2 times a month (17.39%) (10.87%), whereas 18.48% of them increased visits to (almost) every day.

**Figure 7:**
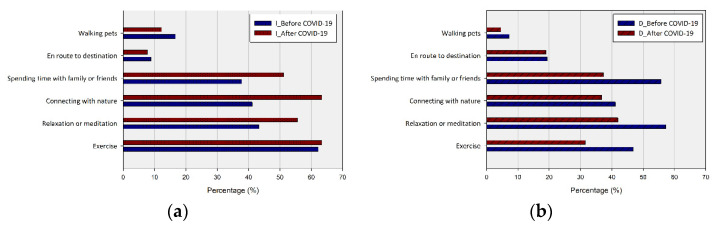
Purpose of UGS visit before and after the COVID-19 outbreak by group with increased visits (**a**) and group with decreased/maintained visits (**b**) to UGS.

The survey on the reason for the change in the UGS use pattern was conducted by dividing them into the use increase group and the maintenance/decrease group. For grouping, the respondents’ visit frequency change was directly selected, and the group with increased visits was 90 people, and the group with a reduced or maintained visit pattern was 124 people. Factors behind the change in utilization patterns were identified through the questions about the purpose of visits before and after COVID-19. The group whose frequency of use increased after COVID-19 had previously visited UGS for the main purpose of exercise (62.22%), relaxation, or meditation (43.33%), but after COVID-19, they visited UGS to experience the natural environment (63.33%) (Figure 7).

**Figure 8:**
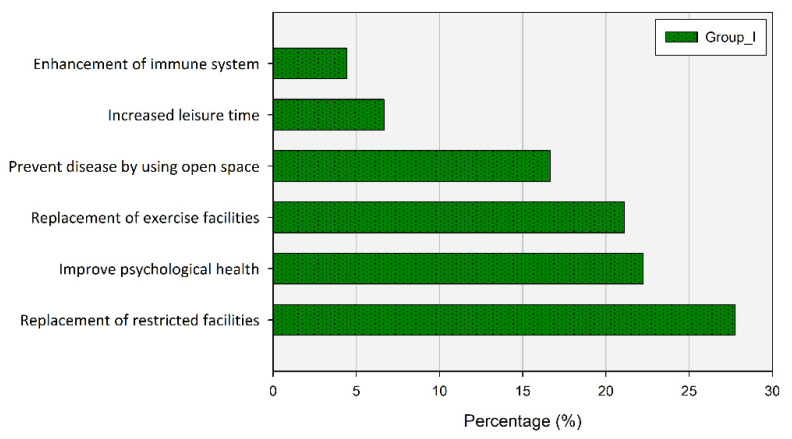
Percentage of respondents according to why UGS visits increased since COVID-19.

In addition, the case of going to UGS to meet family or friends was less at 37.78% before, but it increased to 51.11% after COVID-19 (Figure 8). Their use of UGS increased mainly to replace facilities that prohibited gatherings due to distancing (27.78%) and to promote recovery from depression and for psychological health (22.22%).

**Figure 9:**
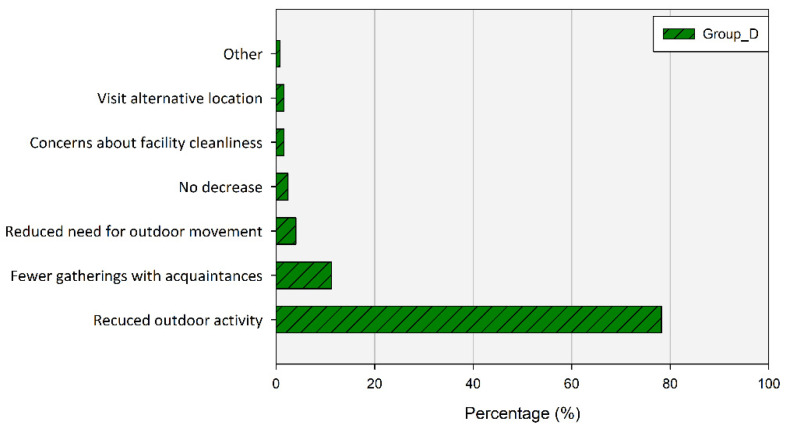
Percentage of respondents regarding why UGS visits decreased since COVID-19.

The decrease/maintain frequency group visited UGS before and after COVID-19 for relaxation or meditation (57.26% to 52.03%) and to meet with family or friends (55.65% to 46.34%) (Figure 7). About 78.26% of this group reduced the number of UGS visits by refraining from going out post-COVID-19 (Figure 9). In other words, it seems that about half of the respondents (49.89%) chose UGS with no restrictions on use when the business hours of the facilities they used were shortened or became unavailable due to government guidelines on distancing due to COVID-19. At the same time, the remarkable increase in the proportion of those who increased their visits to UGS to experience nature and feel psychological stability indicates that they are aware of the CES they obtain from UGS through COVID-19 [58]. Respondents who reduced their UGS visits since the COVID-19 outbreak intentionally tried to reduce their outdoor activities (78.23%) and avoid meeting with family and friends (11.29%). That is, they were judged to be aware of the need to comply with COVID-19 restrictions and social distancing and to minimize contact with others [59].

**Figure 10:**
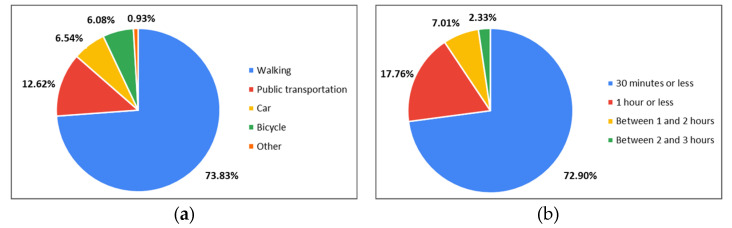
Preferred transportation (**a**) and time (**b**) to visit UGS.

In Figure 10, the suitable transportation and time required for visiting green space are shown. The most preferred means of visiting USG was walking (73.83%), and 72.90% of respondents preferred UGS that could be accessed within 30 min. Preference for means of transportation other than walking differed by age group. For those in their 20 s, the preference for public transportation was 18.07% of respondents in their 20 s, the second highest after walking, but for those in their 30 s and 40 s, cars accounted for more than 12% of respondents in that age group, recording a higher preference than public transportation. Even in the case of people in their 50 s, the preference for public transportation was the second highest after walking, but the preference for walking was the highest at 80.95% among all age groups.

**Figure 11:**
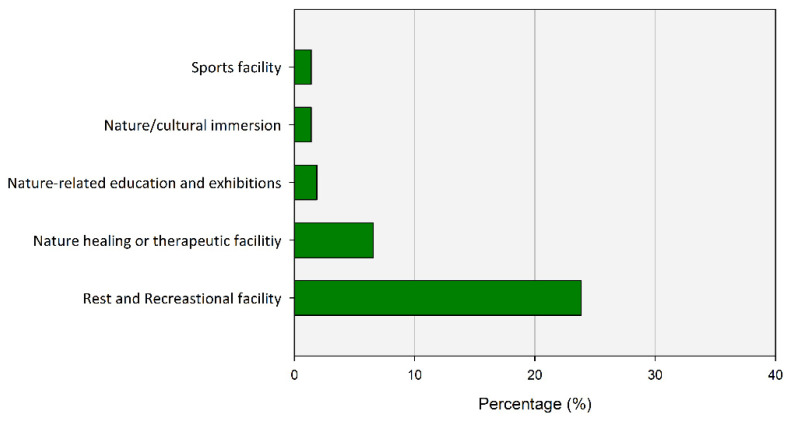
Facilities to encourage the usage of UGS.

Regarding the facilities and services needed to expand and promote the use of UGS in the future, the respondents answered that the expansion of rest and leisure facilities. such as benches and outdoor chairs was needed, with the highest number at about 82.84% (Figure 11). Regarding the facilities and services needed to expand and promote the use of UGS in the future, the respondents answered that the expansion of rest and leisure facilities such as benches and outdoor chairs was needed, with the highest number at about 82.84%. In addition, 55.14% needed natural healing facilities such as forest bathing areas, followed by 35.05% requesting cultural experiences at UGS such as camping grounds.

**Figure 12:**
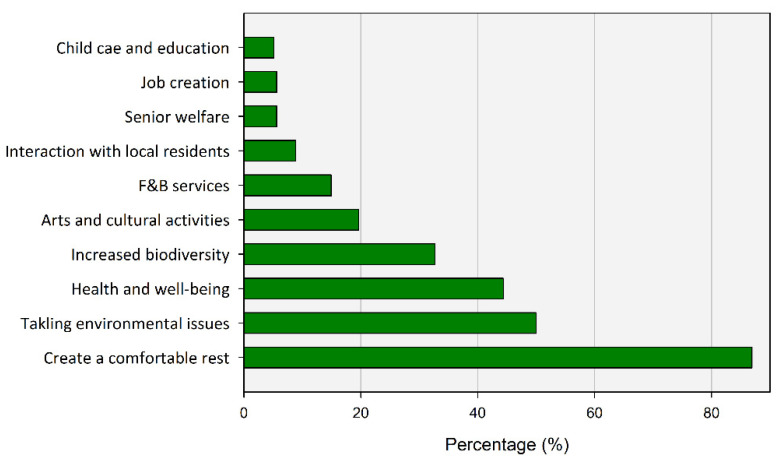
Services to encourage the usage of UGS.

In addition, respondents preferred UGS to develop into a space that provides a more comfortable resting place (86.92%) and alleviates various environmental problems in the city (50%) (Figure 12). In addition, the response that the promotion of the utilization of UGS can be further improved by providing health-friendly services (44.39%) and securing biodiversity (32.71%) followed. Conversely, education, jobs, new connection with local community, and food service were rarely requested from UGS.

## 4. Discussions

The COVID-19 pandemic has had a significant impact on people’s physical and psychological well-being [60,61,62]. From a physical point of view, it mainly caused severe respiratory disease consistently or temporarily [63,64], and from a psychological point of view, national regulations to control the spread of the virus, such as a quarantine and lockdown, resulted in stress, isolation, loneliness, anxiety and depression of people [65,66,67]. Socially, problems such as economic depression and unemployment also occurred [68,69]. In particular, the COVID-19 has been dealt with particularly seriously in cities where the potential for exposure to the virus is high due to high population density [34,70]. As people’s gathering and movement were restricted, the stress and mental health problems of city residents were exacerbated.

However, the awareness and use of the importance of UGS have further highlighted than before COVID-19 in Korea [71], as shown by the high park usage rate even though a strong lockdown policy was implemented in Figure 2. In this study, in the same context, more people than before COVID-19 have come to recognize the importance of UGS as ‘very important, or important’. In particular, among the functions of the UGS, the highest rate stated that the ‘urban environment purification’ was stated at the highest rate, which is in line with previous studies [72,73,74]. It is worth considering in relation to the airborne transmission of coronavirus. Awareness of the function which serves as a barrier to existing air pollutants [75], would have had the effect of improving people’s awareness of UGS during the COVID-19 period. In other words, they may have perceived UGS as a safer place than other places from corona virus transmission [76]. However, it seems that the actual UGS visit frequency itself did not completely match the perception. More than half of all respondents changed their UGS visit patterns due to COVID-19, and in particular, nearly 60% of them reduced their UGS usage frequency. Most of them decreased their UGS visits due to reduced outdoor activities. This is consistent with previous studies showing that green space use declined during the COVID-19 pandemic as people were advised to stay home and avoid crowded places to reduce the spread of the virus [20,77]. However, considering that about 66.15% of users who reduced their use visited UGS at least once a week, they were expected to return when the intensity of lockdown decreases and the magnitude of COVID-19 decreases.

On the other hand, the characteristics of the group that increased UGS use after the outbreak of COVID-19 are noteworthy. It was reported that The increase rate of UGS use was rather high in the group that seldom used UGS before COVID-19. They chose ‘Replacement of restricted facilities’, ‘Improve psychological health’ and ‘Replacement of exercise facilities’ as reasons for the increase in UGS use. In other words, they chose UGS when it became difficult to access the other facilities. In addition, the majority of the groups who used UGS instead of limited facilities responded that they needed more rest facilities such as outdoor chairs and benches to promote UGS use. Through this, the longer duration of time in UGS can be assumed in the group that newly increased UGS visits after COVID-19. This shows a similar pattern to previous study that found the duration of UGS visits became longer overall after COVID-19 [78].

Therefore, in order to and increase the use of UGS by those who have newly realized the value of UGS, it is necessary to install more rest facilities so that many people can stay in UGS for a sufficient amount of time [79,80]. However, there were many users who have increased their visits to UGS to enjoy nature and meditate [32,81], and the overwhelming majority (86.92%) that a comfortable rest space should be created to promote UGS use. Therefore, rest spaces should be provided within the range of not encroaching on the vegetation area as much as possible [82], and considering the high population density, this is possible only when more UGS are secured within the city [73,83]. The increase in UGS is also reasonable considering that the response rate was very high (over 70%) to the UGS being close enough to be within a distance of within 30 minutes by foot from the residence. In conclusion, landscape planning related to the increase of UGS in the city can secure both social consent and sustainability with consistent use by providing sufficient resting space by reflecting the opinions and tendencies of users [80,84,85].

This study had limitations that the survey was conducted on a limited number of subjects for a short period of time. In addition, due to the nature of survey research, questions about the reliability of responses (response bias or self-selection bias) may be raised [86,87]. In other words, there is a limit in that perceptions and attitudes can vary depending on the situation, time, and conditions of survey period [88]. For example, it is difficult to expect that responses collected through the survey will be consistent over time. Therefore, more rigorous and sophisticated survey design and data analysis are required. Nevertheless, the results of the survey conducted in this study show a similar pattern to several previous studies that showed that UGS use increased or decreased under the influence of COVID-19 [38,39,89,90]. Also, the distribution of respondents about the benefits from UGS was found to be similar to previous studies [91,92].

Nevertheless, the implications obtained through this study can support the spread and promotion of UGS in Korea. First, the importance of implementing consumer-oriented policies can be emphasized. South Korea has a green area per capita of 11.6 square meters [93], which is less than that of major global cities such as New York, London, and Singapore [38]. In addition, in order to support carbon neutral policy, central governments such as KFS and MOLIT have established and announced policy goals to expand urban green spaces with the goal of increasing carbon sinks such as urban forests, urban parks, and street trees [94]. However, the local government has not yet specifically presented the area or target area for urban green space expansion. Secondly, investigating and understanding the needs of residents, especially the location and size of new green spaces and related facilities, can increase the effectiveness and sustainability of policies [95,96,97]. Understanding the impact of COVID-19 on the use of urban infrastructure can provide insights for space management and can be used to support the decisions about planning to manage and maintain urban green spaces in the future [98,99]. In addition, it can provide implications on how UGS contribute to physical and mental health individually and social cohesion collectively, and what can be done in the future.

## 5. Conclusions

In this study, we tried to understand the impact of COVID-19 and social distancing on the perceptions and use of UGS through a survey. Understanding the impact of COVID-19 is necessary to promote use and awareness of green spaces. In this process, we found that the patterns of UGS use were polarized depending on individual values when coping with the disease. In addition, citizens’ demand for the expansion of UGS was mainly focused on creating comfortable resting facilities and environments. The fact that UGS facilities that can be accessed within 30 min on foot from residential areas is requested also confirms the basis or necessity for UGS expansion. As this paper found that UGS have not only environmental and ecological values, but also social and health values, there is a high possibility of using UGS to cope with other pandemics or climate crises. With further research and analysis, this white paper can be used as a reference for building green infrastructure as an excellent means of urban planning and design to improve resilience to pandemics and climate change. Finally, it can contribute to the creation of UGS that supports consumer-oriented urban landscape planning and can cope with future disturbances with high resilience.

## Figures and Tables

**Table 1:** Description of the survey.

Personal Characteristics		Frequency	Percentage (%)
Gender	Male	119	55.61
	Female	95	44.39
Age	~19	2	0.93
	20~29	83	38.79
	30~39	44	20.56
	40~49	25	11.68
	50~59	42	19.63
	60~	18	8.41
Occupation	Student	66	30.84
	Office worker	60	28.04
	Service worker	12	5.61
	Education	6	2.80
	Professional	5	2.34
	Technician	41	19.16
	Other	8	3.74
	Unemployed	16	7.48
Workplace (*n* = 213)	Office	117	52.80
	Home	30	16.36
	Combining office and home	66	30.84

**Table 2:** Matrix of changing perceptions about the importance of UGS (unit: person).

After COVID-19						
		Very Important	Important	Neutral	Slightly Important	Not Important at All
Before COVID-19	Very important	73	2	2	0	0
	Important	47	39	5	1	1
	Neutral	15	10	10	2	0
	Slightly important	2	3	0	2	0
	Not important at all	0	0	0	0	0

**Table 3:** Matrix of changing the frequency of UGS utilization (unit: person).

	After COVID-19						
		Daily	Almost Daily	1–2 Times a week	1–2 Times a Month	Rarely	Never
Before COVID-19	Daily	6	5	0	1	0	0
	Almost daily	1	21	6	0	4	0
	1–2 times a week	1	16	48	16	10	1
	1–2 times a month	1	2	14	17	17	2
	Rarely	0	1	4	5	11	3
	Never	0	0	0	0	0	1

## Data Availability

Not applicable.
